# Photosynthetic Characteristics of Three Cohabitated Macroalgae in the Daya Bay, and Their Responses to Temperature Rises

**DOI:** 10.3390/plants10112441

**Published:** 2021-11-12

**Authors:** Xiaohan Shi, Dinghui Zou, Shanshan Hu, Guangming Mai, Zengling Ma, Gang Li

**Affiliations:** 1 School of Environment and Energy, South China University of Technology, Guangzhou 510006, China; xhshi@scut.edu.cn (X.S.); sshu@scut.edu.cn (S.H.); 2 Key Laboratory of Tropical Marine Bioresources and Ecology, South China Sea Institute of Oceanology, Chinese Academy of Sciences, Guangzhou 510301, China; guangingmai@163.com; 3 Southern Marine Science and Engineering Guangdong Laboratory, Guangzhou 519082, China; 4 National and Local Joint Engineering Research Center of Ecological Treatment Technology for Urban Water Pollution, Wenzhou University, Wenzhou 325035, China; mazengling@wzu.edu.cn

**Keywords:** photosynthetic characteristics, temperature rise, *Ulva fasciata*, *Sargassum hemiphyllum*, *Grateloupia livida*

## Abstract

Biochemical compositions and photosynthetic characteristics of three naturally cohabitated macroalgae, *Ulva fasciata*, *Sargassum hemiphyllum* and *Grateloupia livida*, were comparably explored in the field conditions in Daya Bay, northern South China Sea, as well as their responses to temperature rise. Chlorophyll *a* (Chl *a*) and carotenoids contents of *U. fasciata* were 1.00 ± 0.15 and 0.57 ± 0.08 mg g^−1^ in fresh weight (FW), being about one- and two-fold higher than that of *S. hemiphyllum* and *G. livida*; and the carbohydrate content was 20.3 ± 0.07 mg g^−1^ FW, being about three- and one-fold higher, respectively. Throughout the day, the maximal photochemical quantum yield (F_V_/F_M_) of Photosystem II (PS II) of these three macroalgae species decreased from morning to noon, then increased to dusk and kept steady at nighttime. Consistently, the rapid light curve-derived light utilization efficiency (α) and maximum relative electron transfer rate (rETRmax) were lower at noon than that at morning- or night-time. The F_V_/F_M_ of *U. fasciata* (varying from 0.78 to 0.32) was 38% higher than that of *G. livida* throughout the day, and that of *S. hemiphyllum* was intermediate. The superoxide dismutase (SOD) and catalase (CAT) activities in *U. fasciata* were lower than that in *S. hemiphyllum* and *G. livida*. Moreover, the rises in temperature species-specifically mediated the damage (*k*) caused by stressful high light and the corresponding repair (*r*) to photosynthetic apparatus, making the *r*/*k* ratio increase with the rising temperature in *U. fasciata*, unchanged in *S. hemiphyllum* but decreased in *G. livida*. Our results indicate that *U. fasciata* may compete with *S. hemiphyllum* or *G. livida* and dominate the macroalgae community under aggravatedly warming future in the Daya Bay.

## 1. Introduction

Marine macroalgae, including Chlorophyta, Rhodophyta and Phaeophyta, are commonly found in the worldwide coastal regions. They are important in marine ecosystems because they can supply high trophic levels via herbivory or detrital food chains [1], contribute for amount of organic carbon burial [2] and remove surplus nutrients from surroundings [3,4]. Many macroalgae can also provide people with foods [5], medicines [6], biofuels [7] and industrial products [8], as well as be an indicative of environmental health [9]. The macroalgae distribution, growth and productivity are generally mediated by a complex of environmental factors, among which the temperature is particularly important [10,11] because varying temperatures can alter macroalgae enzymes activity, regulating physiological metabolism and ultimately affecting photosynthesis and growth. According to Iñiguez et al. [12], higher temperatures can enhance the activities of the key photosynthetic-involved enzymes, e.g., Ribulose-1,5-bisphosphate carboxylase-oxygenase (RubisCO), thus enhancing the photosynthesis of macroalgae. Such an increased temperature can also neutralize the negative effects of other environmental stressors such as high light [13] and UV-B [14] on photosynthesis. Therefore, moderate increases in temperature have often been observed to stimulate the growth and thus productivity of marine macroalgae [10,13].

Anthropogenic activities, together with climate change are aggravating global warming [15], and over 90% of the excess heat gained by the earth are absorbed by the oceans [16]. Thus, the temperature in surface oceans is predicted to rise by 4 °C by 2100 [17]. Such a temperature increase can even exceed 6 °C under the extremely warming state caused by marine heatwaves [18]. The temperature rise has been observed to lower the coverage of *Durvillaea poha* and largely alter macroalgae diversity in the South Island of New Zealand during the austral hot summer of 2017/18 [18]. Besides large-scale warming, small-scale temperature rises due to thermal effluents from the cooling system of nuclear power station also influence the local marine ecosystem. It is a typical case in the Daya Bay of China, where two nuclear power stations named Daya Bay Nuclear Power Station (2.0 × 10^6^ kW) and Ling-Ao Nuclear Power Station (4.2 × 10^6^ kW) have operated since 1994 and 2002, respectively [19]. The thermal effluents from these two power stations have been recorded to generate an occasional temperature rise of 8 °C, largely altering the species compositions of microbes [20] and phytoplankton [21]. Such a temperature rise must also influence macroalgal physiology and consequently their community in the Daya Bay, although the related studies are scarce.

Daya Bay is a semi-enclosed subtropical bay, and is geographically located in the northern part of the South China Sea. This bay and its adjacent areas have experienced a significant growth since the 1980s [22]. Apart from harbors, petrochemical, plastic, printing and other industries, fish culture has been introduced into this bay since 1985 and has greatly increased in the late 1990s [23]. Growing industrialization, together with marine aquaculture has seriously deteriorated the ecosystem therein [22,24]. Moreover, Daya Bay sustains the high-standing stocks of fishes and benthic animals, as well as rich biodiversity [23]. To date, many studies have been conducted to examine the physical-chemical variables and planktonic features in the Daya Bay [20,21,25,26]. However, a few studies have been carried out on macroalgae although over 200 species are recorded to dwell in this bay [27,28], especially on their photosynthetic characteristics and responses to temperature rise. Therefore, in this study we aimed to characterize (i) photosynthetic characteristics of naturally cohabitated macroalgae, *Ulva fasciata* (Chlorophyta), *Sargassum hemiphyllum* (Phaeophyta) and *Grateloupia livida* (Rhodophyta), three dominant macroalgae species in the Daya Bay; and clarify (ii) how they respond to temperature rise from a photophysiological viewpoint. Probing such the species-specific responses to temperature rise would be helpful to identify which species will be positively affected by warming environments, and which others will be unaffected or even negatively affected, enabling to predict winner and loser species as well as the degree of change in the community in the Daya Bay.

## 2. Results

### 2.1. Field Environments

**Figure 1:**
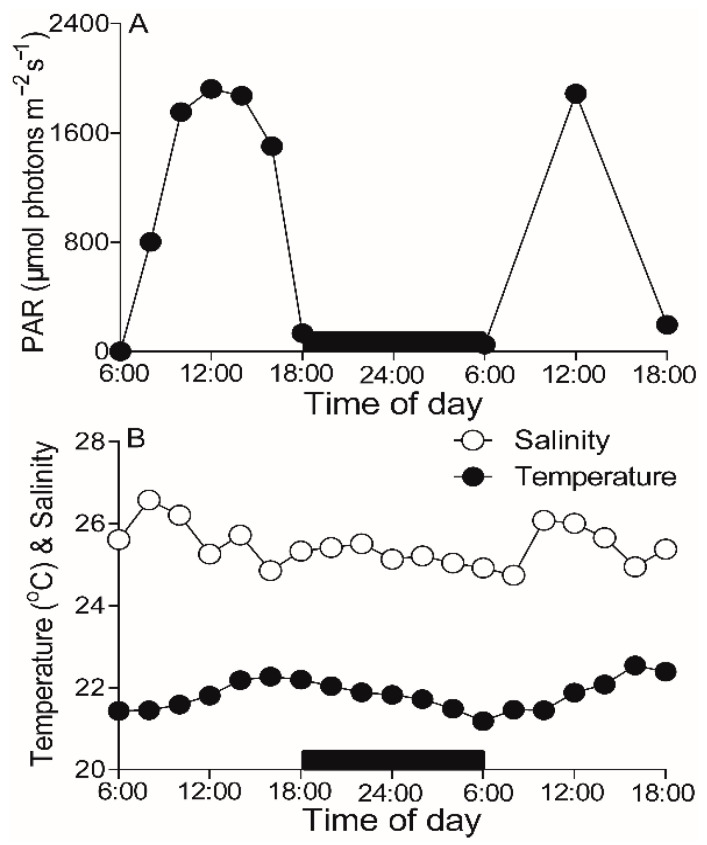
Daily changes of (**A**) atmospheric solar PAR irradiance (µmol photons m^−2^ s^−1^) and (**B**) temperature (°C) and salinity in sampling location during 25–26 March 2021.

During the experimental period of 25–26 March 2021, atmospheric photosynthetically active radiation (PAR) reached a maximum value of ~1900 µmol photons m^−2^ s^−1^ at noontime (Figure 1A). The temperature in the field varied from 21.43 to 22.54 °C, with the minimum and maximum values presented at early morning and dusk, respectively, and the salinity varied from 24.74 to 26.58 (Figure 1B).

### 2.2. Cell Compositions

Basically, the water content and cellular composition of *U. fasciata*, *S. hemiphyllum* and *G. livida* when grown in field condition are shown in Table 1, as well as the antioxidant abilities. Water content in green alga *U. fasciata* was 88.8% ± 2.39%, being approximately 10% lower than that in brown alga *S. hemiphyllum* but similar to red alga *G. livida*. Pigment contents of chlorophyll *a* (Chl *a*) and carotenoids (Car) in *U. fasciata* were 1.00 ± 0.15 and 0.57 ± 0.08 mg g^−1^ FW, being about one- and two-fold higher than that in *S. hemiphyllum* and *G. livida*. Phycoerythrin (PE) and phycocyanin (PC) contents in *G. livida* were 0.16 ± 0.004 and 0.02 ± 0.006 mg g^−1^ FW, respectively. Carbohydrate content in *U. fasciata* was 20.3 ± 0.07 mg g^−1^ FW, about three- and one-fold higher than that in *S. hemiphyllum* and *G. livida*, respectively. Protein content in *U. fasciata* (i.e., 3.19 ± 0.18 mg g^−1^ FW) was similar to *S. hemiphyllum*, but was two-fold higher than *G. livida*. The superoxide dismutase (SOD) and catalase (CAT) activities in *U. fasciata* were 54.2 ± 5.30 and 0.57 ± 0.16 U g^−1^ FW, being significantly lower than the other two species (*p* < 0.05).

### 2.3. Chlorophyll Fluorescence

**Figure 2:**
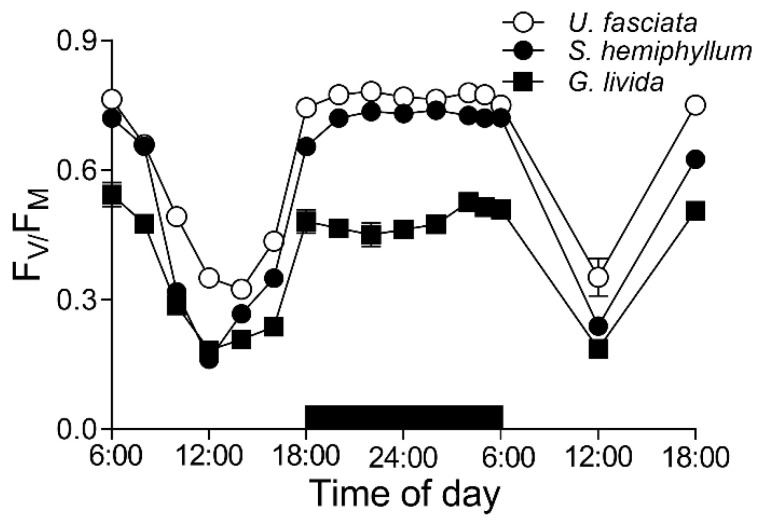
Daily changes of the maximal PS II photochemical quantum yield (F_V_/F_M_) of *U. fasciata*, *S. hemiphyllum* and *G. livida* in field condition. Points show averages of measurements on three independent macroalga thalli, and error bars show the standard deviations (*n* = 3), often within symbols.

**Figure 3:**
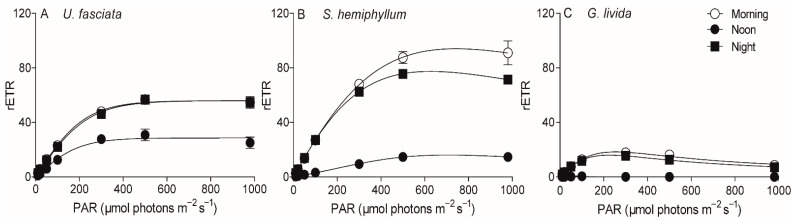
Relative electron transfer rate (rETR) of PS II versus light (µmol photons m^−2^ s^−1^) curves in the time periods of morning, noon and night for (**A**) *U. fasciata*, (**B**) *S. hemiphyllum* and (**C**) *G. livida*. Points show averages of measurements on three independent macroalga thalli, and error bars show the standard deviations (*n* = 3), often within symbols.

To characterize photosynthetic features of these three cohabitated macroalgae, we continuously tracked their maximal photochemical quantum yield (F_V_/F_M_) of Photosystem II (PS II), an indicator of photosynthetic potential, throughout a 36-h time period (i.e., two days and one night) with 2-h interval (Figure 2). The F_V_/F_M_ of *U. fasciata* decreased from 0.76 ± 0.01 to 0.35 ± 0.02 from morning to noon, then increased to 0.77 ± 0.04 at dusk and kept steady during the nighttime. Throughout the daytime, there was ~38% higher in the F_V_/F_M_ values of *U. fasciata* than that of *G. livida* (i.e., varying from 0.53 ± 0.01 to 0.18 ± 0.02); and *S. hemiphyllum* showed the intermediate F_V_/F_M_ values (i.e., varying from 0.74 ± 0.01 to 0.16 ± 0.02). Consistently, the rapid light curves (RLCs) of these three macroalgae species exhibited the same daily changes, with the relative electron transfer rate (rETR) being significantly lower at noontime than that at morning- or night-time (*p* < 0.05) (Figure 3); the RLC-derived light utilization efficiency (α) and maximum rETR (rETRmax) were also lower at noontime than in the morning or nighttime (Table 2). Furthermore, the saturation irradiance (E_K_) showed no significant change in the morning, noon and nighttime in *U. fasciata* (i.e., 223 ± 14.6 µmol photons m^−2^ s^−1^), but varied greatly in both *S. hemiphyllum* and *G. livida* (Table 2).

### 2.4. Effects of Temperature Rise

**Figure 4:**
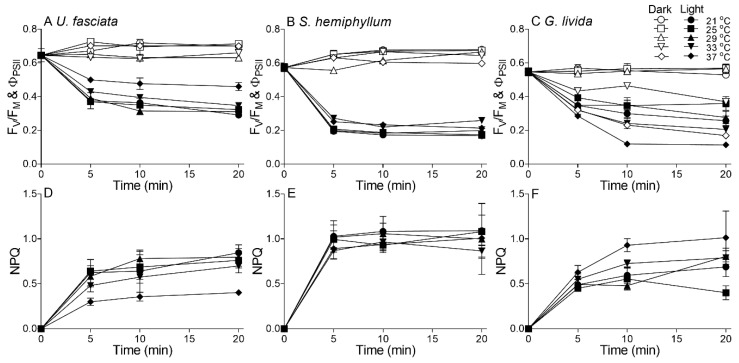
Time-series changes of PS II photochemical quantum yield ((**A**–**C**); Φ_PS II_ under light, or F_V_/F_M_ under dark) and non-photochemical quenching ((**D**–**F**); NPQ) of (**A**,**C**) *U. fasciata*, (**B**,**D**) *S. hemiphyllum* and (**C**,**F**) *G. livida* under temperatures of 21 (field condition), 25, 29, 33 and 37 °C under the dark and local-noon light (800 µmol photons m^−2^ s^−1^) conditions. Points show averages of measurements on three independent macroalga thalli, and error bars show the standard deviations (*n* = 3), often within symbols.

In the current study, we measured the short-term time course of PS II function of all these three macroalgae species under five temperature treatments to probe the effects of acute temperature rise (Figure 4). Considering the light intensity mediates algal responses to other environmental factors including temperature [10,11,13,14], here we comparatively tracked the time-series changes of photosynthetic efficiency (i.e., PS II photochemical quantum yield) in the dark (F_V_/F_M_) and under the local noon light state (Φ_PS II_) (Figure 4A–C). In the dark, the photosynthetic efficiency (i.e., F_V_/F_M_) of the three macroalgae species showed no significant change with time among all temperature treatments; in the light state however, the photosynthetic efficiency (i.e., Φ_PS II_) markedly decreased with exposure time, and the decreased degree differed greatly among the three macroalgae species and the temperature treatments. 

**Figure 5:**
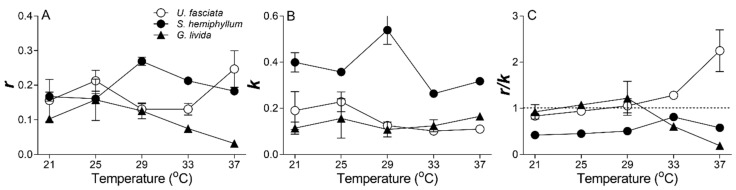
Changes of the photosynthetic capacity (Φ_PS II_) versus time-derived rate constants of repair ((**A**); *r*, min^−1^) and damage ((**B**); *k*, min^−1^), and (**C**) *r*/*k* ratio with temperature rise for *U. fasciata*, *S. hemiphyllum* and *G. livida*. Points show averages of measurements on three independent macroalga thalli, and error bars show the standard deviations (*n* = 3), often within symbols.

Non-photochemical quenching (NPQ), an indicatiion of light stress, also showed a different varying trend with increasing temperature among the three species. In *U. fasciata*, the NPQ decreased with increasing temperature, indicating higher temperature may lessen the high-light pressure (Figure 4C); in contrast, the NPQ increased in *G. livida* with increasing temperature, indicating an aggravated function of high temperature upon the light stress (Figure 4E). The NPQ in *S. hemiphyllum* showed no obvious variation among different temperatures (Figure 4D). Furthermore, the rate constant of repair (*r*) estimated from Kok model [29] scattered among the five temperatures in *U. fasciata*; while the *r* showed an optimum value at 29 °C in *S. hemiphyllum*, but a decreasing trend with increasing temperature in *G. livida* (R^2^ = 0.55, *p* < 0.05) (Figure 5A). Similarly, the rate constant of light-induced damage (*k*) scattered in *U. fasciata* and showed an optimum value at 29 °C in *S. hemiphyllum* as well (Figure 5B). The ratio of *r* to *k* (*r/k*) exhibited no significant change from low to intermediate temperatures (Figure 5C); from medium to high temperatures however, it increased in *U. fasciata* (R^2^ = 0.89, *p* < 0.05) but decreased in *G. livida* (R^2^ = 0.98, *p* < 0.05), indicating a species-specific response to the combined light and thermal stress.

## 3. Discussion

Most organisms including macroalgae on the earth exhibit diel rhythms in behavior or/and physiology [30]. We also found such a diel change in photosynthetic performance of green, brown and red macroalgae, but with species-specific among *U. fasciata*, *S. hemiphyllum* and *G. livida* in the Daya Bay. Moreover, green alga *U. fasciata* had higher photosynthetic potential than brown alga *S. hemiphyllum* and red alga *G. livida* in the field condition and the temperature rise strengthened the resistance of *U. fasciata* to the local noontime stressful light by promoting repairability over light-caused damage, indicating the *U. fasciata* may compete with *S. hemiphyllum* and *G. livida* and dominate the community under aggravatedly warming future in the Daya Bay.

Maximal PS II photochemical quantum yield (F_V_/F_M_), an indicator of macroalgae photosynthetic potential [10,25,31], showed a clear “noonday inhibition” due to the local noon stressful light condition (Figure 2); and as such, the light-caused decrease-degree in *U. fasciata* was less (i.e., the slope of F_V_/F_M_ against PAR, S = −2.16 × 10^−4^, *p* < 0.01) than that in *S. hemiphyllum* (i.e., S = −2.52 × 10^−4^, *p* < 0.01), but more than *G. livida* (i.e., S = −1.74 × 10^−4^, *p* < 0.01). It is common that the F_V_/F_M_ decreases under the high light condition [32,33], because, the stressful light is generally believed to inactivate the PS II reaction center [34]. Consistently, the diel pattern of F_V_/F_M_ showed an opposite trend to solar irradiation (Figure 1A). Moreover, the great decrease of F_V_/F_M_ at noontime, as well as light utilization efficiency (α, Table 2), can be considered as the reversible photoinhibition, rather than the photodamage if considering the F_V_/F_M_ recovered completely after the removal of light stress in late afternoon. Furthermore, the F_V_/F_M_ of *U. fasciata* was higher than that of *S. hemiphyllum* or *G. livida* (Figure 2), consistent with the results derived from the comparisons of six macroalgae species in a coastal area of Gouqi Island, China [31]. 

Morphologically, *Ulva* species has larger surface area (SA) that satisfies them to obtain resources like light, Ci or nutrients and to maintain higher photosynthetic capacity, although the larger SA also means a suffering of higher hydrodynamic forces that may cause detrimentally mechanical damages [35]. On the other hand, the green algae including *U. fasciata* contain Chl *a*/*b* as main light-harvesting pigments for photosynthesis; while the brown (e.g., *S. hemiphyllum*) and red algae (e.g., *G. livida*) respectively contain additional Chl *c* and phycobilin as auxiliary channels to obtain light source [36,37]. Such the differences in the pigment composition and content (Table 1) may also attribute to the difference in photosynthetic capacity among these macroalgae species, which is supported by the lower F_V_/F_M_ in red algae (Figure 2) that is associated with the presence of phycobilisome in the PS II light-harvesting complex [38]. In nature, physio-chemical environments are well known to affect the PS II activity (e.g., F_V_/F_M_) of macroalgae [25,38]; however, our results showed no significant effects of varying temperature and salinity upon the F_V_/F_M_ (*p* > 0.05), indicating the change range of physio-chemicals within a day may not reach the threshold.

Under the stressful high light, the photosynthetic potential of *U. fasciata* declined less when compared to that of *S. hemiphyllum* and *G. livida* (Figure 1 and Figure 4), indicating its more light resistance. Generally, the excess light energy absorbed by the light-harvesting complex can generate more reactive oxygen species within cells [39], that could destroy photosynthetic apparatus and reduce photosynthetic capacity. Accordingly, different macroalgae species may have evolved different strategies to adapt field light, including morphological and biochemical etc. [40,41,42]. It is known that the higher SA to volume (V) ratio prevails the thalli of *U. fasciata* than that of *S. hemiphyllum* and *G. livida*, which means the *U. fasciata* is more susceptible to high light as the “package effect” is lower [43,44]. It is in contrast to our finding that *U. fasciata* was more light-resistant than the other two macroalgae species (Figure 4), so the possibility of morphological causes can be eliminated in this case. On the other hand, *Ulva* species can excrete polysaccharides outside the cells and thus form a film on the thalli surface especially under stressful high light [40], which may protect the thalli from the harmful light and help to maintain the higher photosynthetic capacity. The *Ulva* species can also dissipate the absorbed excess light energy through a fast non-photochemical quenching (NPQ) driven by lutein cycle, which may lower the potential photooxidative damage and protect the thylakoid membrane from irreversible damage [45,46]; while this mechanism does not work in red algae including *G. livida*, as they have no lutein within cells [47]. However, our data did not support it with varying NPQ values among *U. fasciata*, *S. hemiphyllum* and *G. livida* (Figure 4D–F), and the underlying mechanisms need to be studied further.

Furthermore, most plants can take advantage of repair as the main way to alleviate the light-caused photoinhibition [45,48]. According to Kok [29], the light-induced PS II damage (*k*) and corresponding repair (*r*) occur simultaneously within cells, and the *r*/*k* ratio can be used to indicate the dynamics between the damage and repair processes. The damage processes of PS II are photochemically driven, and are thus less temperature-dependent, while the repair processes of e.g., the new protein resynthesis and transportation are enzyme-involved and more temperature-dependent [49]. Therefore, the rising temperature may have shifted the balance towards repair rather than the damage of *U. fasciata* and strengthened its resistance to the stressful light, as indicated by increasing *r/k* ratio (Figure 5C). Moreover, higher cellular proteins and carbohydrates concentrations that prevail in *U. fasciata* (Table 1) may also help to maintain its higher repair capacity if considering the proteins are usually the major components of all kinds of key enzymes and substrates that involve in the repairing process [50,51]. Such a positive effect of temperature rise has also been detected in green algae *Ulva bulbosa* and *Ulva clathrata* [52]. In red alga *G. livida*, the *r/k* ratio was lower under higher temperatures (Figure 5C), indicating more susceptibility to stressful light. Such a high temperature may have exceeded the optimal value of *G. livida* and deactivated the enzymes involved in the repairing process (Figure 5A), thus leading to more inhibition of photosynthesis (Figure 2 and Figure 3). Moreover, *G. livida* contains large amount of phycobilisomes, the temperature-sensitive proteins [53], to serve for light harvesting. The increased temperature may thus have caused degradation of these phycobilisomes, and declined its photosynthetic ability. Finally, the light level at local noontime in the Daya Bay may have severely photodamaged the PS II of brown alga *S. hemiphyllum* and as such, the light-caused damage cannot be overcome by the repair process. Therefore, this makes the *r/k* ratio to be lower than 1 throughout the temperature range (Figure 5). More resistance to the high light and thermal stress may make *U. fasciata* compete with *S. hemiphyllum* and *G. livida* and dominate the community in the Daya Bay.

## 4. Materials and Methods

### 4.1. Study Area and Experimental Protocol

On 25–26 March 2021, we conducted the in situ experiment on a fish-raft 500 m offshore with Chlorophyta *Ulva fasciata*, Phaeophyta *Sargassum* *hemiphyllum* and Rhodophyta *Grateloupia livida* in the Daya Bay (114°31′ E, 22°44′ N), Shenzhen, China. Daya Bay covers an area of ~550 km^2^, and has depths of 5 m to 18 m and an annual mean temperature of ~22 °C [50,54]. All these three macroalgae are dominating species in this bay and matured during the study period, and naturally cohabitated around the fish-raft, as well as on the rocky seabed. The sampling depth was ~1.0 m, with local noon PAR level (10:00 a.m.–14:00 p.m.) of ~800 μmol photons m^−2^ s^−1^ according to [55].

Throughout the experimental periods, we measured the field’s physical and chemical environments every 2 h. At the same time, we measured the photochemical quantum yield (F_V_/F_M_) of Photosystem II (PS II) of all these three adult macroalgae species from field condition to detect their photosynthetic characteristics; and we also measured the rapid light curves (RLC) of all these macroalgae species at morning-(8:00 a.m.), noon-(12:00 p.m.) and night-time (20:00 p.m.) as described below. For each measurement, we used three individual thalli of each species.

To detect the effects of acute temperature rises, we measured time-series changes of the PS II photochemical quantum yields under five temperatures in the dark and under the local noontime sunlight condition, because we considered the light mediates the physiological responses of algae to the temperature rise.

### 4.2. Environmental Factors Measurements

Every 2 h, atmospheric PAR irradiation at the sampling site was monitored with a PAR sensor (US-SQS/L, ULM-500, Walz, Germany), and the temperature and salinity in the field were measured with a multi-parameter water quality monitor Sonde (YSI 6600, Yellow Springs Instruments, Yellow Springs, OH, USA).

### 4.3. Chlorophyll Fluorescence Measurements

Every 2 h, 2–3 cm of the thalli was cut off from each of triplicate healthy mother thalli grown in field condition. Then, the chlorophyll fluorescence of the thalli was measured using a portable chlorophyll fluorometer (AquaPen-C 100, Photon Systems Instruments, Prague, Czech Republic) after 15 min of dark acclimation. The maximal PS II photochemical quantum yield (F_V_/F_M_) was calculated as below, with the measured maximal fluorescence (F_M_) under a saturation light pulse (~3000 μmol photons m^−2^ s^−1^, 0.6 s) and the baseline fluorescence (F_O_) under a weak modulated measuring light; and the effective PS II quantum yield (Φ_PS II_) was calculated with the maximal fluorescence (F_M_′) under saturation light and instantaneous fluorescence (Ft) under light status. The F_V_/F_M_ and Φ_PS II_ values were calculated [56] as:

F_V_/F_M_ = (F_M_ − F_O_)/F_M_; Φ_PS II_ = (F_M_′ − F_t_)/F_M_′

The relative electron transport rate (rETR) of PS II was measured under seven different actinic lights (PAR, µmol photons m^−2^ s^−1^), for 60 s exposure at each light level to obtain the rapid light curve (RLC). The rETR was estimated [56] as:

rETR = Φ_PS II_ × 0.5 × PAR × 0.84

where the 0.5 and 0.84 indicate the absorbed light energy being equally allocated to PS II and PS I and the light energy absorbed efficiency, respectively.

The RLC-derived photosynthetic parameters, i.e., light utilization efficiency (α), saturation irradiance (E_K_) and maximal rETR (rETRmax) were calculated [57] as:

rETR = PAR/(a × PAR^2^ + b × PAR + c)

α = 1/c; rETR_max_ = 1/[b + 2 × (a × c)^1/2^]; E_K_ = c/[b + 2 × (a × c)^1/2^]

where a, b and c are adjusted parameters.

To estimate the effects of acute temperature rise, the thalli of all three macroalgae species were collected in the morning (8:00 a.m.–10:00 a.m.), cut into 2–3 cm pieces and dark-incubated for 10 min [10] in the tanks where the temperature was maintained at 21 (field temperature), 25, 29, 33 and 37 °C with a thermostatted bath. On the 20-cm top of one tank, the light intensity, close to local-noon sunlight was supplied by a lamp (Sylvania 17W T8 4100K) (i.e., light treatment), and another tank was covered with aluminum foil (i.e., dark treatment). The Φ_PS II_ (measured under 800 μmol photons m^−2^ s^−1^ actinic light) and F_V_/F_M_ (in the dark) under each temperature were tracked at the time-points of 0, 5, 10 and 20 min, respectively. The non-photochemical quenching (NPQ) was calculated as:

NPQ = F_M0_/F_Mt_′ − 1

where F_M0_ and F_Mt_′ represent the maximal fluorescence at time t0 and t.

The rate constant of the light-caused damage (*k*, min^−1^) and that of corresponding repair (*r*, min^−1^) to photosynthetic apparatus were estimated using Kok model [29] as:

P/P_0_ = *r*/(*r* + *k*) + *k*/(*r* + *k*) × e^(−(*r* + *k*) × t)^

where P_0_ and P represent the Φ_PS II_ at time t0 and t.

If using a, b and c to represent *r*/(*r* + *k*), *k*/(*r* + *k*) and *r* + *k*, this exposure-response curve can be reformed as:

P/P_0_ = a + b × e^(−c × t)^

So, the *r* and *k* can be calculated as:

*r*= a × c; *k* = b × c

### 4.4. Cellular Compositions Measurements

To determine the cellular composition of *U. fasciata*, *S. hemiphyllum* and *G. livida,* the algal thalli were collected from the field condition, and transported to laboratory within a dark-carrying bucket. After returning to laboratory, the water content, pigments, carbohydrate and proteins, as well as antioxidant activity were determined with 3 individual biological replicates for each species as bellow:

For water content, 3–5 g pre-weighted fresh thalli of each species was dried overnight in an oven (100 °C). After cooling, the dried thalli were re-weighted; and the water content was estimated with the weight difference between fresh- and dried-thalli divided by the fresh weight.

For pigments content, 0.1 g fresh thalli were transferred into 10 mL absolute methanol, ground with quartz sands (HF-24, Hefan Instrument Co., Ltd., Shanghai, China), and extracted overnight at 4 °C in the dark. After 10-min centrifugation (5000× *g*) at 4 °C, the optical absorption spectra of supernatant were scanned with spectrophotometer (UV-1800, Shimadzu, Kyoto, Japan); and chlorophyll *a* (Chl *a*) and carotenoids (Car) concentrations (mg g FW^−1^) were calculated [58] as:

Chl *a* (mg g FW^−1^) = [16.29 × (A_665_ − A_750_) − 8.54 × (A_652_ − A_750_)] × 10 mL × 10^−3^ g µg^−1^ × FW^−1^

Car (mg g FW^−1^) = [7.6 × (A_480_ − A_750_) − 1.49 × (A_510_ − A_750_)] × 10 mL × 10^−3^ g µg^−1^ × FW^−1^

where A_750_, A_665_, A_652_, A_510_ and A_480_ represent the absorption at 750, 665, 652, 510 and 480 nm wavelengths, and FW indicates fresh weight.

To quantify phycocyanin (PC) and phycoerythrin (PE) in *G. livida*, 0.2 g fresh thalli were extracted in 10 mL-0.1 M phosphate buffer (pH 6.8) and ground. After 10-min centrifugation (5000× *g*, 4 °C), the supernatant was scanned with the spectrophotometer. The PC and PE contents were calculated [59] as:

PC (mg g FW^−1^) = [(A_618_ − A_645_) − 0.51 × (A_592_ − A_645_)] × 0.15 × 10 mL × FW^−1^

PE (mg g FW^−1^) = [(A_564_ − A_592_) − 0.20 × (A_455_ − A_592_)] × 0.12 × 10 mL × FW^−1^

where A_645_, A_618_, A_592_, A_564_ and A_455_ represent the absorption at 645, 618, 592, 564 and 455 nm wavelengths.

For carbohydrate content, 0.2 g fresh sample was homogenized with 2 mL distill water and some sands using the multi-sample tissue grinder. The mixture was then transferred into a 5-mL tube and incubated in boiled water for 10 min. After centrifuging (5000× *g*) for 10 min at 4 °C, the carbohydrate in supernatant was quantified with a carbohydrate assay kit (A045-1-1, Nanjing Jiancheng Biological Engineering Company, Nanjing, China) following the manufacturer’s protocol, with an anthrone-sulfuric acid method [60].

To measure the protein content, 0.1 g of the fresh sample was homogenized with 2 mL–0.1 M phosphate buffer (pH 6.8) and some sands using the multisample tissue grinder at 4 °C. After centrifuging (5000× *g*) for 10 min at 4 °C, the protein in the supernatant was quantified using a soluble protein assay kit (A045-2-1, Nanjing Jiancheng Biological Engineering Company, China) according to manufacturer’s protocol, with a Coomassie Brilliant Blue colorimetric method [61]. After this, superoxide dismutase (SOD) and catalase (CAT) activities in the protein solution were determined with the assay kits (A001-1-1 for SOD, and A007-1-1 for CAT, Nanjing Jiancheng Biological Engineering Company, China) following the protocol of the kits supplied by the manufacturer.

### 4.5. Data Analysis

The mean and standard deviations (mean ± sd) were presented in figures, and two-way Repeated Measures ANOVA, paired *t*-test and one-way ANOVA with Bonferroni post-tests (Prism 5, GraphPad Software) were used to detect the significant difference among different treatments or species, with confidence level of 0.05.

## 5. Conclusions

In this study, we found the biochemical compositions differed greatly among the three cohabitated macroalgae *U. fasciata*, *S. hemiphyllum* and *G. livida*, as well as their photosynthetic characteristics throughout the day. Green alga *U. fasciata* contained one- to three-fold higher Chl *a*, carotenoids and carbohydrate than brown alga *S. hemiphyllum* and red alga *G. livida* and exhibited a larger daily variation in photosynthetic potential. For *U. fasciata*, the temperature rise alleviated the photoinhibition caused by the local noontime high light through promoting the repairability over light-caused damage of PS II. While for *G. livida*, the temperature rise aggravated such the photoinhibition through lowering the repair ability. Rising temperature showed a limited effect on the photoinhibition of *S. hemiphyllum*, as well as the balance between repair and damage. Our results indicate that the green algae (e.g., *U. fasciata*) may compete with brown (e.g., *S. hemiphyllum*) and red algae (e.g., *G. livida*) and dominate the community in the future if the aggravated warming continues in the Daya Bay.

## Figures and Tables

**Table 1:** Water content (%), biochemical compositions (mg g^−1^ FW) of Chl *a*, carotenoids (Car), phycoerythrin (PE), phycocyanin (PC), carbohydrate and proteins, and superoxide dismutase (SOD) and catalase (CAT) activities (U mg^−1^ protein) of *U. fasciata*, *S. hemiphyllum* and *G. livida* grown in field condition. Numbers show the mean and standard deviations (mean ± sd) (*n* = 3); and different letters on top-right of number indicate the significant difference among three algae species (*p* < 0.05).

Cell Compositions	*U. fasciata*	*S. hemiphyllum*	*G. livida*
Water (%)	88.8 ± 2.39 ^a^	96.8 ± 1.16 ^b^	87.9 ± 2.27 ^a^
Chl *a* (mg g^−1^ FW)	1.00 ± 0.15 ^a^	0.62 ± 0.02 ^b^	0.34 ± 0.02 ^c^
Car (mg g^−1^ FW)	0.57 ± 0.08 ^a^	0.19 ± 0.01 ^b^	0.12 ± 0.01 ^c^
PE (mg g^−1^ FW)	--	--	0.16 ± 0.004
PC (mg g^−1^ FW)	--	--	0.02 ± 0.006
Carbohydrate (mg g^−1^ FW)	20.3 ± 0.07 ^a^	5.19 ± 0.67 ^b^	9.45 ± 0.09 ^c^
Protein (mg g^−1^ FW)	3.19 ± 0.18 ^a^	3.04 ± 0.16 ^a^	1.20 ± 0.20 ^b^
SOD (U g^−1^ FW)	54.2 ± 5.30 ^a^	68.4 ± 3.58 ^b^	61.7 ± 0.50 ^c^
CAT (U g^−1^ FW)	0.57 ± 0.16 ^a^	0.88 ± 0.05 ^b^	1.46 ± 0.08 ^c^

**Table 2:** The rapid light curve (RLC)-derived light utilization efficiency (α), saturation irradiance (E_K_, µmol photons m^−2^ s^−1^), and maximum relative electron transport rate (rETRmax) of *U. fasciata*, *S. hemiphyllum* and *G. livida* grown in field condition, measured at morning, noon and nighttime. Numbers show the mean and standard deviations (mean ± sd) (*n* = 3); and different letters on top-right of number indicate the significant difference among three measured time-periods (*p* < 0.05).

Parameters	*U. fasciata*			*S. hemiphyllum*			*G. livida*		
	Morning	Noon	Evening	Morning	Noon	Evening	Morning	Noon	Evening
α	0.28 ± 0.003 ^a^	0.13 ± 0.015 ^b^	0.26 ± 0.007 ^c^	0.29 ± 0.014 ^a^	0.03 ± 0.002 ^d^	0.29 ± 0.011 ^a^	0.16 ± 0.011 ^e^	--	0.16 ± 0.026 ^e^
EK	210 ± 11.0 ^a^	239 ± 33.4 ^a^	223 ± 5.46 ^a^	331 ± 37.9 ^b^	607 ± 83.4 ^c^	264 ± 6.2 ^d^	116 ± 21.6 ^e^	--	99.0 ± 19.4 ^e^
rETR_max_	58.0 ± 2.96 ^a^	31.2 ± 3.88 ^b^	57.7 ± 0.08 ^a^	94.4 ± 7.62 ^d^	16.3 ± 1.17 ^e^	77.7 ± 1.74 ^f^	18.3 ± 2.46 ^e^	--	15.9 ± 0.61 ^e^

## Data Availability

The data presented in this study are available within the article.
